# Errors during Gene Expression: Single-Cell Heterogeneity, Stress Resistance, and Microbe-Host Interactions

**DOI:** 10.1128/mBio.01018-18

**Published:** 2018-07-03

**Authors:** Christopher R. Evans, Yongqiang Fan, Kalyn Weiss, Jiqiang Ling

**Affiliations:** a Department of Microbiology and Molecular Genetics, Medical School, University of Texas Health Science Center, Houston, Texas, USA; b MD Anderson Cancer Center UTHealth Graduate School of Biomedical Sciences, Houston, Texas, USA; National Cancer Institute

**Keywords:** mistranslation, phenotypic heterogeneity, protein synthesis, stress response

## Abstract

Gene expression has been considered a highly accurate process, and deviation from such fidelity has been shown previously to be detrimental for the cell. More recently, increasing evidence has supported the notion that the accuracy of gene expression is indeed flexibly variable. The levels of errors during gene expression differ from condition to condition and even from cell to cell within genetically identical populations grown under the same conditions. The different levels of errors resulting from inaccurate gene expression are now known to play key roles in regulating microbial stress responses and host interactions. This minireview summarizes the recent development in understanding the level, regulation, and physiological impact of errors during gene expression.

## IMPORTANCE

Gene expression has been considered a highly accurate process, and deviation from such fidelity has been shown previously to be detrimental for the cell. More recently, increasing evidence has supported the notion that the accuracy of gene expression is indeed flexibly variable.

## INTRODUCTION

**FIG 1:**
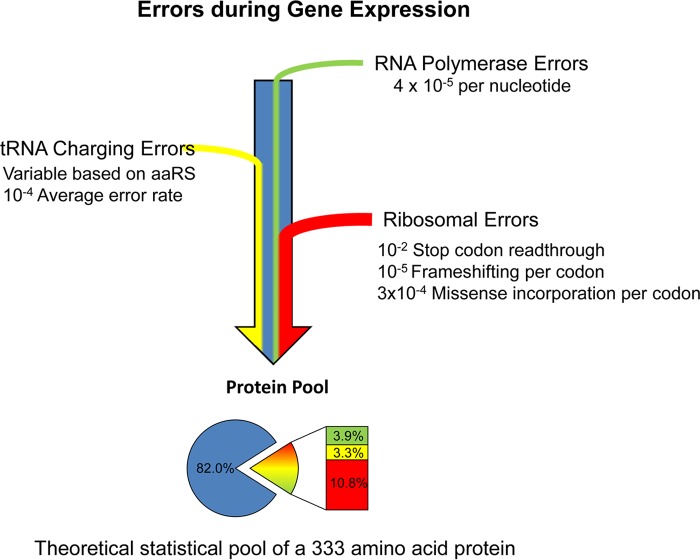
Errors during gene expression. Nonheritable errors during gene expression can come from transcription and translation. For a coding gene with around 300 to 400 codons, approximately 10% to 20% of the proteins made contain at least one error, such as missense incorporation, frameshifting, or stop codon readthrough. This fraction of erroneous proteins significantly increases when the error rates per codon are increased by genetic and environmental factors, leading to a statistical proteome containing very diverse protein variants encoded by the same gene. aaRS, aminoacyl-tRNA synthetase.

Gene expression is a fundamental process in all living cells and controls the accurate flow of genetic information from DNA to RNA to protein. To ensure the accuracy of gene expression, extensive substrate selection and proofreading mechanisms are utilized at each step during DNA replication, transcription, and translation (1–3). For instance, aminoacyl-tRNA synthetases selectively pair each amino acid with the correct tRNAs and utilize editing functions to hydrolyze mismatched aminoacyl-tRNAs (4, 5). Despite such conserved quality control mechanisms, the fidelity of gene expression is not fixed under all conditions (Fig. 1). Genetic and environmental changes can substantially increase the levels of errors during gene expression. In this minireview, we summarize recent developments in our understanding of the prevalence, regulation, and physiological impact of gene expression errors, with a focus on microbial organisms.

## FIDELITY OF GENE EXPRESSION

### Transcriptional fidelity.

Accurate transcription of DNA into mRNA is essential for the transfer of genetic information to the protein synthesis machinery. Despite the clear role that transcriptional accuracy must play in gene expression, transcriptional error rates have been estimated to reach 10^−5^ errors per nucleotide (2). Traditionally, transcriptional error rates are determined using *in vitro* transcription by RNA polymerases or *in vivo* reporters (6). Several recent studies have used high-throughput RNA sequencing with improved fidelity during cDNA synthesis and sequencing to determine global transcriptional error rates in multiple bacteria (7). The transcriptional error rates measured by RNA sequencing match the *in vitro* error rates on the order of 10^−5^. Interestingly, transcriptional error rates are similar between extracellular Escherichia coli and endosymbiotic bacteria despite the striking differences in genome sizes and growth conditions (7). In addition, the transcriptional error rate is not affected by growth stages or nutrient sources (7). It appears that transcriptional fidelity is optimized during evolution to resist perturbation by environmental cues. Only a few genetic factors have been identified to control transcriptional fidelity; these include transcription elongation factors GreA and GreB (2, 8) and stringent response regulator DksA (9, 10). Due to their transient nature, the errors generated during transcription are thought to have less of an impact than the DNA replication errors that accumulate over generations. However, increased transcriptional errors have been shown to significantly affect the molecular heterogeneity (noise) of gene expression (discussed below) (10–12).

### Translational fidelity.

Compared with transcriptional errors, the overall amino acid misincorporation (missense) rate in the protein is an order higher at approximately 10^−4^ to 10^−3^, largely due to imperfections of aminoacyl-tRNA synthesis and ribosomal decoding (4, 13–16). Other types of translational errors, such as stop codon readthrough and frameshifting, could occur more frequently in bacteria and eukaryotes at 10^−2^ (17–22). It is worth pointing out that under certain circumstances, recoding resulting from translational readthrough or frameshifting depends on the context of mRNA sequences and can lead to production of alternative functional proteins and provide a selective advantage during evolution (20, 23). Such context-dependent recoding events are generally not considered gene expression errors and are beyond the scope of this minireview.

Translational errors have been measured using radiolabeled amino acids (24, 25), enzyme reporters (14, 26–29), mass spectrometry (19, 28, 30), ribosome profiling (31, 32), and fluorescent reporters (18, 21, 33–37). All those assays indeed measure the overall error rates in gene expression, but, given that translation is much more error-prone than DNA replication and transcription, such results serve as a good estimation for the error rate during translation. Despite the technical advancement and growing interest, the picture of the actual rates of different translational errors still remains blurry. It is now increasingly clear that translational fidelity is affected by genetic and environmental factors (3, 38–40), and the same type of translational error may differ from cell to cell (18, 34). To allow accurate quantitation of different translational errors in cells under native growth conditions, further advances in the availability of sensitive reporters and in mass spectrometry technology are much needed.

## HETEROGENEITY OF GENE EXPRESSION

Bacterial populations are comprised of millions of clonal cells. Despite the genetic similarity between these cells, individual cells within a population exhibit a wide variety of physiological phenotypes (41). Nearly every aspect of bacteria physiology, including shape, size, growth rate, motility, and stress tolerance, has some level of heterogeneity (noise) within a population. Many of the mechanisms that can lead to population heterogeneity have been reviewed elsewhere (41), and variation in gene expression has been shown to be a critical contributor to the heterogeneity among cells. More-recent work has revealed how different aspects of gene expression, from initiation of transcription to production of a polypeptide, are heterogeneous between single cells in a population.

### Transcriptional heterogeneity.

The better-understood aspect of noisy gene expression is transcriptional heterogeneity. Experimental evidence for gene expression noise within a population was first revealed in bacterial cells (42, 43). Ozbudak et al. showed that the expression levels of a fluorescent protein differ from cell to cell within a population of genetically identical Bacillus subtilis cells (43). Using two fluorescence reporters controlled by identical promoters in E. coli, Elowitz et al. found that promoter activity is heterogeneous among cells and is stochastic within the cell, particularly when the transcription level is low (42). Both of those studies used protein fluorescence as the readout for gene expression, and the overall heterogeneity of fluorescence intensity reflected the cumulative noise from transcription, mRNA degradation, translation, protein degradation, and fluorophore maturation. To specifically study transcriptional noise, a breakthrough came from the use of MS2-green fluorescent protein (MS2-GFP) to directly count the number of stable mRNA molecules carrying the MS2 binding sites in E. coli (44). Subsequent studies revealed that transcription initiation does not occur continuously but rather as bursts (45, 46). Variations in promoter activity are large contributors to variations in single-cell gene expression. In 2012, a study characterized the heterogeneity of every known promoter in E. coli and found that different promoters show different levels of heterogeneity in a population (47). Some categories of promoters, such as stress response promoters, are noisier than others (47). Heterogeneity of gene expression was initially thought to be a consequence of the stochastic nature of molecular interactions (42). However, recent analyses of the evolution of synthetic promoters *de novo* revealed that the heterogeneity of promoter expression is low by default (48). This finding indicates that the high levels of heterogeneity seen in some promoters may have evolved as a beneficial mechanism. Future investigations into the regulation of promoter heterogeneity and evolution of these systems may provide insights into the role and benefits of transcriptional heterogeneity in bacterial populations.

Transcriptional heterogeneity has been directly tied to phenotypic heterogeneity in bacterial populations. The mechanisms by which gene expression heterogeneity can influence bacterial physiology have been previously reviewed (41, 49). Recently, a report showed that the levels of heterogeneity itself are regulated and can influence the fitness of a bacterial population under stress (50). In that work, Carey et al. showed that an E. coli population responds to changes in O_2_ levels by altering the heterogeneity of a signal transduction system without changing the population mean. This indicates that the mechanisms controlling transcriptional heterogeneity can be regulated independently of the average transcription level and highlights the importance of further studies of single-cell gene expression dynamics.

Despite our improving knowledge of overall transcriptional heterogeneity, the noise of transcriptional errors is poorly understood due to technical challenges. Interestingly, Herman and colleagues have shown that increasing transient transcriptional errors by deleting *greAB* or *dksA* genes can alter the stochastic switching frequency of gene expression (10–12). This leads to bistable feedback loops and heritable phenotypic changes. In future studies, it will be intriguing to investigate whether variations of transcriptional errors among individual cells directly correlate with bistable gene expression.

### Translational heterogeneity.

Compared to transcriptional noise, the posttranscriptional heterogeneity of gene expression has not been extensively studied. This is primarily due to technical limitations because the noise from transcription is difficult to filter out. However, recently developed reporters can account for changes in single-cell transcription and have provided insights into how variability in posttranscriptional processes may affect the proteome and cell physiology in single cells.

Like changes in transcription, changes in translational rates ultimately have a significant effect on the protein levels in a cell. As such, variations in the overall translational rate in a cell or variations in affinity for ribosome binding sites could contribute to the cell heterogeneity within a population. Despite these similarities between transcription and translation, much less is known about the mechanisms and impact of translation on population heterogeneity. Early work used single fluorescent reporters to determine how translation initiation and codon context affect heterogeneity (43, 51). Those pioneering studies revealed that altering translation initiation and elongation perturbs the overall gene expression noise. However, signals of single fluorescent reporters are heavily influenced by transcriptional levels, making it difficult to fully examine the contribution of translation to the overall gene expression heterogeneity (52).

The recent development of dual-fluorescence reporters to measure the heterogeneity of translational fidelity has been a step forward toward our understanding of posttranscriptional gene expression noise. These reporters use a control fluorescence protein that is translationally fused to a second fluorescence protein in order to normalize differences in transcription and translation initiation in single cells and have enabled the quantification of ribosomal missense errors (34–36), stop codon readthrough (18), and frameshifting events (18, 21, 37). The concept of dual reporters that are translationally fused originated from a dual-luciferase system (14, 29, 53). Compared to luciferase reporters, dual-fluorescence reporters allow quantitation of translational errors within single cells using either fluorescence imaging or flow cytometry. It needs to be noted that in performing quantitation using such reporters, the signal or activity of the first reporter should not be much affected by the fusion. Should this not be the case (i.e., if the first reporter is affected by the fusion), one remedy would be to introduce a linker that allows the reporters to rapidly split following translation (54).

Studies using the dual-fluorescence reporters have revealed that translational errors within single cells are noisy and can lead to phenotypic heterogeneity (18, 34). In mycobacteria, misincorporation of glutamate at glutamine codons occurs at a frequency of approximately 1% and can cause phenotypic resistance to rifampin by producing drug-resistant variants of the target protein RpoB (34, 55). In an elegant study, Javid and colleagues used fluorescence-activated cell sorting to show that misincorporation rates differ from cell to cell and that the subpopulations of cells with high misincorporation rates survive better in the presence of rifampin (34). We have recently developed a dual-fluorescence reporter system to measure stop-codon readthrough and frameshifting and have used fluorescence microscopy to quantitatively demonstrate that such translational errors are heterogeneous among single cells in E. coli (18) and Salmonella enterica serovar Typhimurium (K. Weiss and J. Ling, unpublished results). We further used time-lapse microscopy to show that cells with increased readthrough of the UGA stop codon exhibit an increased rate of recovery from the stationary phase compared to cells with a low readthrough rate (18). Those studies suggest that, in addition to gene expression levels, other factors of gene expression (such as translational fidelity) can play a crucial role in cell physiology at the single-cell level.

## GENE EXPRESSION ERRORS AND MICROBIAL STRESSES

Microorganisms constantly experience changing environments and quickly reprogram gene networks to adapt to stress conditions (40, 56, 57). Increasing evidence supports the notion that gene expression errors play a critical role in sensing and responding to various environmental stresses. For example, carbon starvation increases translational frameshifting (58) and stop codon readthrough (22) in E. coli, and oxidative stress increases amino acid misincorporation errors (28, 59, 60). To learn more about the mechanisms and conditions that cause alteration of translational fidelity, readers are referred to two excellent recent reviews (39, 40).

**FIG 2:**
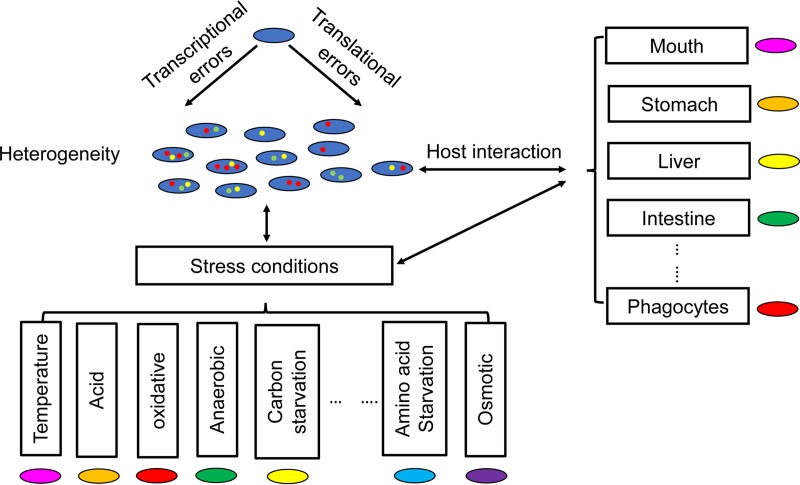
Single microbial cells in a population of genetically identical cells display heterogeneity in gene expression errors due to stochasticity of transcription and translation. The mean and heterogeneity of errors can be influenced by environmental factors, such as different stresses. Errors in gene expression in turn make microbial cells better adapted or less well adapted to stress and host environments.

Gene expression errors are regulated by environmental factors but, conversely, also affect adaptation of microbes to environmental conditions, such as stresses (Fig. 2). As discussed above, transcriptional and translational errors are significant sources of molecular noise and lead to a statistical proteome with mixed protein variants encoded by the same gene, providing phenotypic diversity that allows the microbial population to survive and thrive. For example, phenotypic mutations resulting from an error-prone RNA polymerase with a 20-fold increase in transcriptional errors promote evolution of β-lactam resistance (62). Transcriptional errors also lead to heritable phenotypic changes as a consequence of activation of bistable switches that regulate important pathways, including metabolic gene and cellular differentiation pathways (11, 49).

Inaccuracy in the translation machinery appears to have more diverse and profound effects on microbial fitness and stress resistance than transcriptional errors (Table 1). Accumulation of translational errors has been shown to cause proteome destabilization, growth defects, and even cell death (63–66). Reports of recent work from the laboratory of M. Ibba showed that editing deficiencies in phenylalanyl-tRNA synthetase (PheRS), which cause misincorporation of Tyr and *meta*-Tyr at Phe codons, attenuate amino acid stress response in bacteria and yeast (67, 68). In E. coli, starvation of Phe leads to accumulation of uncharged tRNA^Phe^, which activates transcription of Phe biosynthesis gene *pheA*. PheRS editing deficiencies cause mischarging of tRNA^Phe^ and repress transcription of *pheA* (67). Similarly, uncharged tRNA activates an amino acid starvation response in yeast through the Gcn2/Gcn4 pathway, and PheRS editing deficiency decreases activation of Gcn2p (68). On the other hand, editing defects in aminoacyl-tRNA synthetases benefit bacterial growth when the cognate amino acid is limited in abundance and the mischarged amino acid is abundant (69, 70). Various translational errors have also been reported to improve resistance against antimicrobial, oxidative, and heat stresses (summarized in Table 1). For instance, translational errors lead to resistance against oxidative stress in bacteria and yeasts, but those effects likely occur via distinct mechanisms (71–73).

**TABLE 1:** Effects of translational errors on microbial stress resistance

Translational error(s)	Stress condition(s)	Organism(s)	Fitness	Reference(s)
Ile → norvaline	Amino acid starvation	Escherichia coli	Gain	69
Ile → Val	Amino acid starvation	Acinetobacter baylyi	Gain	70
Phe → *meta*-Tyr	Amino acid starvation	Escherichia coli	Loss	67
Phe → Tyr	Amino acid starvation	Saccharomyces cerevisiae	Loss	68
Gln → Glu; Asn → Asp	Antibiotics	Mycobacteria	Gain	34, 55
Ile → Val	Antibiotics	Escherichia coli	Gain	82
Met misincorporation	Antibiotics	Escherichia coli	Gain	83
CUG codon ambiguity	Antifungal drugs	Candida albicans	Gain	81
Arg → canavanine	Heat stress	Saccharomyces cerevisiae	Gain	84, 85
CUG codon ambiguity	Oxidative and osmotic stresses	Saccharomyces cerevisiae	Gain	72
Global mistranslation	Oxidative stress	Escherichia coli	Gain	27, 71
Stop codon readthrough	Various stresses	Saccharomyces cerevisiae	Gain/loss	73, 86
Ile → Val	Sporulation	Bacillus subtilis	Loss	87

## TRANSLATIONAL ERRORS AND MICROBE-HOST INTERACTIONS

Microbial pathogens must adapt to diverse host environments during infection by triggering specific stress responses and expression of virulence genes (57). In addition to stress resistance, translational errors have also been shown to play a critical role in microbe-host interactions. Modifications of the 16S rRNA gene, such as methylation modifications, are important for maintaining accuracy in translation initiation (74). Deficiencies in 16S rRNA methylation have been reported to decrease virulence in Staphylococcus aureus due to increased sensitivity to oxidative stress (75, 76). Restrictive mutations in the ribosomal protein RpsL enhance translational fidelity (77) and decrease survival of S. enterica serovar Typhimurium in mice (78, 79), suggesting that moderate levels of translational errors in the wild-type bacteria are important for adaption to host environment. In the fungal human pathogen Candida albicans, 97% of CUG codons are translated as Ser and 3% as Leu (80). Bezerra et al. showed that C. albicans strains with increased levels of Leu incorporation at CUG positions cause enhanced host immune response (81), and it has been suggested that CUG ambiguity has evolved to potentially enhance cell surface variability (80). Our overall understanding of how translational fidelity impacts microbe-pathogen interactions is only at the beginning stage, and much work needs to be performed to elucidate the roles of various translational errors in the invasion and survival of different microbial pathogens within hosts.

## CLOSING REMARKS AND OUTLOOK

Studies in the past couple of decades have shown that fidelity in gene expression is dynamic and highly regulated. Increased errors during gene expression have various effects on cell fitness and stress responses. However, not all errors are the same, and different types of errors can elicit very different responses even in the same organism. For the most part, the mechanisms by which different gene expression errors lead to physiological changes are not clearly understood and await future investigations. Recent developments in fluorescence reporters have provided a high-throughput platform to determine the error rates during gene expression and now empower us to track various errors under the native growth conditions experienced by microbes (e.g., within biofilms or hosts) at the single-cell and population levels. Translational errors appear to be heterogeneous among single isogenic microbial cells. In future work, it will also be intriguing to understand how such noise in gene expression leads to heterogeneity in diverse microbial phenotypes.
